# Optically Transparent Carbon–Silicon Nitride Windows for Correlative Structural and Electrochemical Analysis of Nanomaterials

**DOI:** 10.1021/acs.analchem.5c01403

**Published:** 2025-05-23

**Authors:** Sasha E. Alden, Oluwasegun J. Wahab, Lingjie Zhang, Kelly L. Vernon, Baixu Zhu, Kathleen O. Bailey, Xingchen Ye, Lane A. Baker

**Affiliations:** † Department of Chemistry, 14736 Texas A&M University, College Station, Texas 77843, United States; ‡ Department of Chemistry, 1771 Indiana University, Bloomington, Indiana 47405, United States

## Abstract

We report an optically transparent carbon electrode–silicon nitride (OTCE–SiN_*x*_) window platform fabricated through clean-room microfabrication techniques. A wafer-scale fabrication process was implemented, which enabled a batch preparation of 2925 windows (100 × 100 μm) in user-friendly configurations. Application of OTCE–SiN_*x*_ windows is demonstrated in the high-resolution structural and nanoscale electrochemical characterization of nanocrystals by transmission electron microscopy (TEM) and electrochemical scanning probe microscopy. High-resolution TEM (HR-TEM) and aberration-corrected high-angle annular dark-field TEM (HAADF-TEM) were achieved for nanocrystals supported on the OTCE–SiN_*x*_, with minimal background electron scattering or interference from the OTCE–SiN_*x*_. Additionally, the stability of OTCE–SiN_*x*_ under prolonged voltammetric cycling was investigated. Scanning electrochemical cell microscopy (SECCM) revealed uniform nanoscale electrochemistry. Correlative electrochemical and electron microscopy on OTCE–SiN_*x*_ was demonstrated for the hydrogen evolution reaction (HER) at clusters of sub-10 nm Au nanospheres.

## Graphical abstract

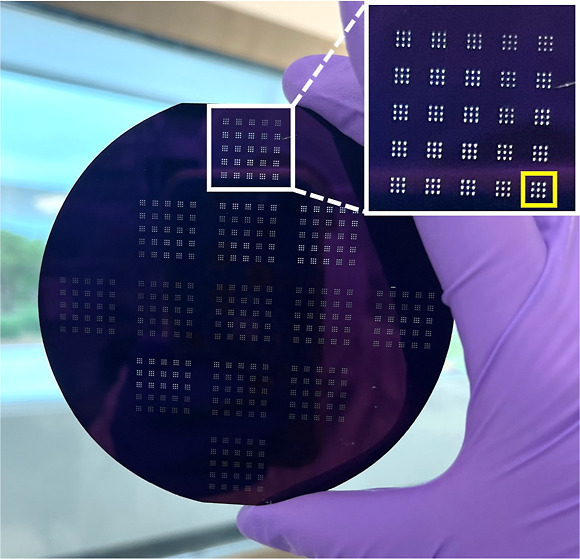

## Introduction

A direct understanding of the structure–activity relationship of nanomaterials is critical to advance the design and applications of nanoparticle-based catalysts.^1,2^ Toward this goal, correlative multimodal electrochemical microscopies^3^ provide a route to unraveling structure–activity at the nanoscale.^4^ Electrochemical data from scanning electrochemical cell microscopy (SECCM) can be directly correlated with identical-location spectroscopic and surface characterization methods such as optical microscopy (OM), scanning/transmission electron microscopy (SEM/TEM), atomic force microscopy (AFM), Raman spectroscopy, and electron backscatter diffraction.^5^ In this respect, SECCM has emerged as a prevalent method to study reactions such as hydrogen evolution reaction (HER),^6,7^ the oxygen evolution reaction (OER),^8–10^ and CO_2_ reduction,^11^ on nanomaterial systems typically ranging from 10s to 100s of nanometers in size, ideal for SEM and AFM characterization.^11–22^

To bridge the gap between the dimensions of materials used in model studies and nanomaterials used in industrial catalytic processes, where critical size regimes are 1–10 nm,^23–25^ routine correlative electrochemical microscopy methods to study sub-10 nm nanoparticles are desirable. Correlative SECCM-TEM is suitable for this challenge, with significant benefits in linking electrochemical activity to structural and compositional details down to the atomic scale, but is experimentally difficult compared to correlative SECCM-SEM or SECCM-AFM. Further, correlative SECCM-TEM demands substrates with specific properties, such as appropriate thinness and minimal electron scattering, without compromising nanoscale electrochemical performance. This has led to fewer SECCM-TEM studies.^9,10,26,27^ Note that correlative SECCM-TEM is distinct from identical location-transmission electron microscopy (IL-TEM),^28,29^ where identical locations of nanoparticle-loaded electrodes are imaged before and after macroscale electrochemical reactions to study early stages of electrodeposition or corrosion and particle morphology changes.^28–34^ Unlike SECCM-TEM, IL-TEM measures the electrochemical response from the entire sample, weakening the correlation between structure and function, especially for samples with significant heterogeneity.

In previous correlative SECCM-TEM studies that involved nanomaterials, commercial TEM grids (typically a Cu or Au grid covered with a thin carbon layer) were used.^8,9,35,36^ The frames of these grids are flexible, making them significantly challenging to handle as substrates for electrochemical imaging experiments and to translate to SEM, TEM, and OM instruments. Additionally, the nanoscale electrochemical activity of the carbon layer (typically, holey or lacey carbon films) remains largely unexplored, and there have been reports of degradation or delamination of commercial TEM grids during use.^37,38^ Other SECCM-TEM reports utilized specialized equipment^10^ or involved complex procedures, such as focused ion beam (FIB) milling, to extract thin sections of dense materials for scanning transmission electron microscopy (STEM) analysis.^26,27^ Thus, a critical need for more robust and versatile support electrodes that allow a seamless transfer of nanomaterial samples between (nanoscale) electrochemical measurements and TEM characterization exists. Recent advances in ion-milled boron-doped diamond electrodes,^32,37^ which are suitable for both high-resolution TEM (HR-TEM) and electrochemical applications, represent a step toward addressing this challenge.

Microelectro-mechanical system (MEMS) chips with silicon nitride windows used for in situ TEM applications are promising platforms that can be adapted for correlative SECCM-TEM applications. The microfabricated devices are equipped with a SiN_*x*_ layer suitable for TEM viewing and Si frames for structural support. In addition to their sturdy nature, which aids convenient handling for electrochemical imaging and transfer to other techniques, these platforms are highly customizable, a feature that has been significantly exploited in the literature for different designs, sizes, and number of windows.^39–43^ However, the existing commercially available MEMS devices offer limited utility for correlative electrochemical imaging applications, as they are typically designed for one-time use for in situ TEM, where a series of high-magnification and small area images are acquired. For correlative SECCM-TEM applications, multiple and wider windows are required to accommodate numerous spot measurements and several scans that span tens of microns. Another desirable feature is the suitability of the substrate for multiscale experiments, where nanomaterial samples are deposited on a large (millimeter) scale, and areas with suitable particle distribution or desired features can be located for nano-to-microscale electrochemical imaging experiments. Further, the nanoscale electrochemical attributes of the support electrode are critical in these experiments and underscore the need for modified TEM grids or chips specifically designed for electrochemical imaging.

Here, the fabrication and characterization of transparent, conductive, and electrochemically homogeneous windows for correlative SECCM and TEM analysis are presented. Arrays of TEM windows were fabricated at the wafer scale by first generating SiN_*x*_ windows, with Si(100) fractures to derive multiple grids.^44^ The SiN_*x*_ support provides a more robust structure than that of the commercial Cu or Au grids. Subsequent pyrolysis of the photoresist on the SiN_*x*_ windows generated optically transparent carbon electrodes (OTCEs).^45,46^ The choice of pyrolyzed photoresist films (PPFs)^47,48^ for OTCE is motivated by low roughness, suitable conductivity, and chemical robustness,^49^ as recently detailed at larger scales for correlative SECCM-OM.^50^ Further, while TEM supports based on graphene layers are good for imaging as they offer minimal background electron scattering,^39,51–53^ electrochemical kinetics at monolayer and few-layer graphene are sluggish.^54–56^ OTCE–SiN_*x*_ windows were characterized by high-resolution TEM and electrochemical imaging. SECCM voltammetric mapping of ruthenium hexaammine reduction revealed ideal electrode kinetics. Atomically resolved images of Au nanospheres were successfully obtained with OTCE–SiN_*x*_ TEM grids. SECCM was used to study HER activity at clusters of ∼8 nm Au nanospheres deposited on an OTCE–SiN_*x*_ TEM grid by SECCM. While isolated single nanoparticle electrocatalysis data is not realized, the results presented sufficiently highlight the methodology of using the fabricated SiN_*x*_ window for correlative SECCM-TEM.

## Materials and Methods

### Microfabrication of OTCE–SiN_*x*_ Windows

Fabrication of OTCE–SiN_*x*_ TEM windows was carried out at the wafer scale using 200 μm thick, 100 mm diameter, (100) double-sided polished (DSP) silicon wafers (University Wafer, Boston, MA) (Figure 1 Step 1). Low-pressure-chemical vapor deposition (LP-CVD, Tytan 3600) deposited 60 nm of low-stress SiN_*x*_ on both sides of the wafer (Step 2). Photoresist (AZ1512) and photolithography were used to transfer the TEM window pattern to one side of the wafer (Step 3). The patterned side of the wafer underwent an isotropic etch via reactive ion etching (RIE, PlasmaLab 100, Oxford) with CF_3_H, removing SiN_*x*_ from exposed areas. A subsequent wet anisotropic etch of Si by 27 wt % KOH solution was carried out for ∼3 h at 75 °C to create SiN_*x*_ windows (Step 7). A thin layer of the S1813 resist diluted with propylene glycol monomethyl ether acetate (1:5) was applied to the window side of the wafer by spin coating at 3k RPM for 45s using a nonvacuum chuck (Laurell Technologies) (Step 8). Pyrolysis of the resist occurred in a tube furnace under a reducing atmosphere (6% H_2_ 94%N_2_) at 1000 SCCM flow at a ramp rate of 15 °C/min to reach a temperature of 1000 °C and is held there for 60 min before ramping down to room temperature. Wafers remained in the chamber for the entire duration of the ramp and cool-down process. Wafer-scale fabrication produced 13 chips (∼1 × 1 in.), each containing 25 3 × 3 mm TEM grids, with each TEM grid containing 9 100 × 100 μm SiN_*x*_ windows. Chips and individual TEM grids are designed with partially etched lines facilitating easy breakout, allowing for measurements to be made at either the chip or TEM grid level (See Figure S1 of the Supporting Information).

**Figure 1:**
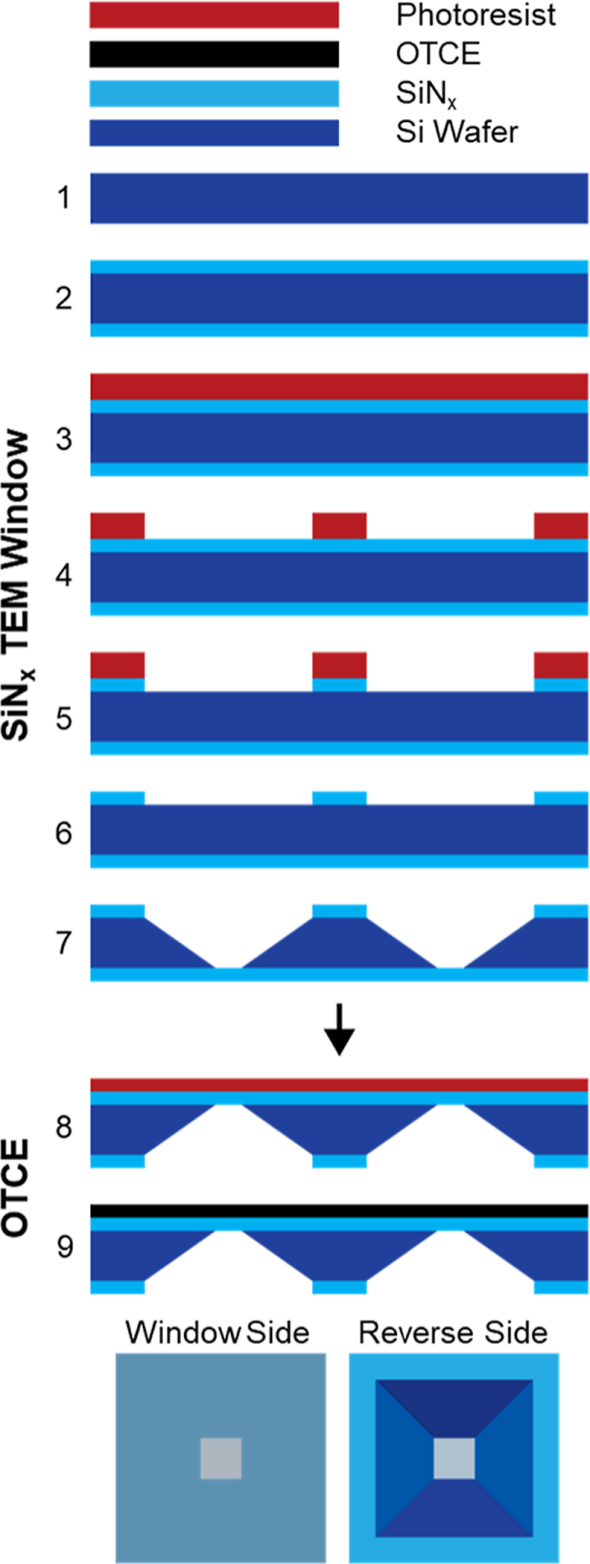
Scheme of the optically transparent carbon electrode (OTCE)–SiN_*x*_ window microfabrication.

### Macroscale Cyclic Voltammetry (CV) OTCE−SiN_*x*_ Grids

To prepare OTCE–SiN_*x*_ for macroscale CVs, OTCE–SiN_*x*_ was fixed onto a cover glass (Microscope Cover Glass cat. no. 12-544D, Fisher Scientific Co.) with double-sided tape. Conductive Ag epoxy (AA-Duct 904, Atom Adhesives) was painted onto a corner of the OTCE–SiNx and up the cover glass and then set to dry overnight. Wire (AWG20 Hook Up Wire Kit, Plusivo) was connected to the conductive Ag epoxy by copper tape (Copper Conductive Tape, 1/4″, Electron Microscopy Sciences). The sample was masked with Kapton tape (Thomas Scientific) with a 1 mm diameter hole toward the center of the OTCE–SiN_*x*_, exposing 4 windows to create a well-defined electroactive surface area. A photograph of the final assembly is shown in Supporting Information Figure S4a. All macroscale voltammetry was performed with a CHI660C potentiostat (CH Instruments, Texas).

### Imaging and Sample Preparation

Optical images of OTCE–SiN_*x*_ TEM grids were collected by an Eclipse LV150N (Nikon) in brightfield mode. TEM imaging was carried out with a JEOL 1200 EX TEM (JEOL) for low-resolution imaging and Titan Themis 300 S/TEM (Thermo Fisher) for high-resolution TEM (HR-TEM) and HAADF-STEM. Nanoparticles were dropcast on TEM grids in diluted solutions, followed by solvent washing to remove extraneous ligands. Au nanocubes were diluted 10× with water before dropcasting. Ligands were removed by successive immersion first in methanol then water (30 s each). Au nanospheres were diluted 65× with toluene before dropcasting. Ligands were removed by successive immersions in methanol (60 s) and water (30 s).

### Scanning Electrochemical Cell Microscopy

SECCM was engaged to perform the electrochemical mapping of OTCE–SiN_*x*_ windows (Figure 5). Nanopipettes were pulled from either quartz capillaries (initial dimensions 1.0 mm outer diameter (O.D.), 0.5 mm inner diameter (I.D.), final tip diameter ∼50 nm), using a laser puller (P-2000, Sutter Instruments), or borosilicate capillaries (1.0 mm O.D., 0.58 mm I.D., final tip diameter ∼800 nm), using a filament puller (P-1000, Sutter Instruments). Details of representative ca. 50 and 800 nm nanopipet tips used in this report are included in the Supporting Information. Additional scanning parameters and hardware are described in the Supporting Information.

## Results and Discussion

OTCE–SiN_*x*_ windows were fabricated in a 9-step process beginning with a DSP 100 mm diameter Si wafer in a clean room environment as illustrated in Figure 1. Silicon nitride membranes are derived from modifications to well-established fabrication processes.^44,57^ LP-CVD was used to deposit 60 nm films of low-stress SiN_*x*_ (Figure 1, step 2), where the ratio of Si is higher than that of stoichiometric nitride (Si_3_N_4_). LP-CVD films exhibited a refractive index of 2.07, indicative of properly formed low-stress nitride. Following nitride deposition, photolithography and RIE of exposed SiN_*x*_ on the reverse side of the wafer was used to define location of windows and scoring lines to facilitate later chip/grid release (Steps 3–6). Anisotropic etch of Si in heated KOH removed Si from the reverse side of the wafer to reveal SiN_*x*_ windows (Step 7, Supporting Information Figure S2). A dilute photoresist was spin coated onto the window side of the wafer, followed by pyrolysis in a tube furnace in a reducing atmosphere (6% H_2_/94% N_2_) at 1000 °C (Steps 8–9). The resultant OTCE films were ∼22 nm thick, as measured by AFM (Figure S3). Together with the 60 nm SiN_*x*_ membrane, the OTCE–SiN_*x*_ window is ∼82 nm thick. Characterization of the prepared OTCE–SiN_*x*_ with AFM and x-ray photoelectron spectroscopy (XPS) is presented in Figure S3. A sheet resistance for the OTCE film of 2.3k Ohm/□ (read as kilo-ohm per square) was measured by a 4-point probe. While the sheet resistance constitutes a relatively large internal resistance, the low magnitude of current passed makes typical SECCM measurements effectively immune to the ohmic drop.^50^ Optical (Figure 2a–d) and low-magnification TEM (LM-TEM) (Figure 2e–g) images of representative OTCE–SiN_*x*_ windows show wrinkles (Figure 2b), likely caused by thermal stress encountered during pyrolysis (wrinkles proved inconsequential in subsequent electrochemical or electron microscopy characterization). Each chip (Figure 2c) contains 25 TEM grids with 9 windows per grid. Each window is 100 × 100 μm (Figure 2d). One wafer produces 325 TEM grids with 2925 OTCE–SiN_*x*_ windows (Figure S1), making this a relatively high-throughput fabrication process.

**Figure 2:**
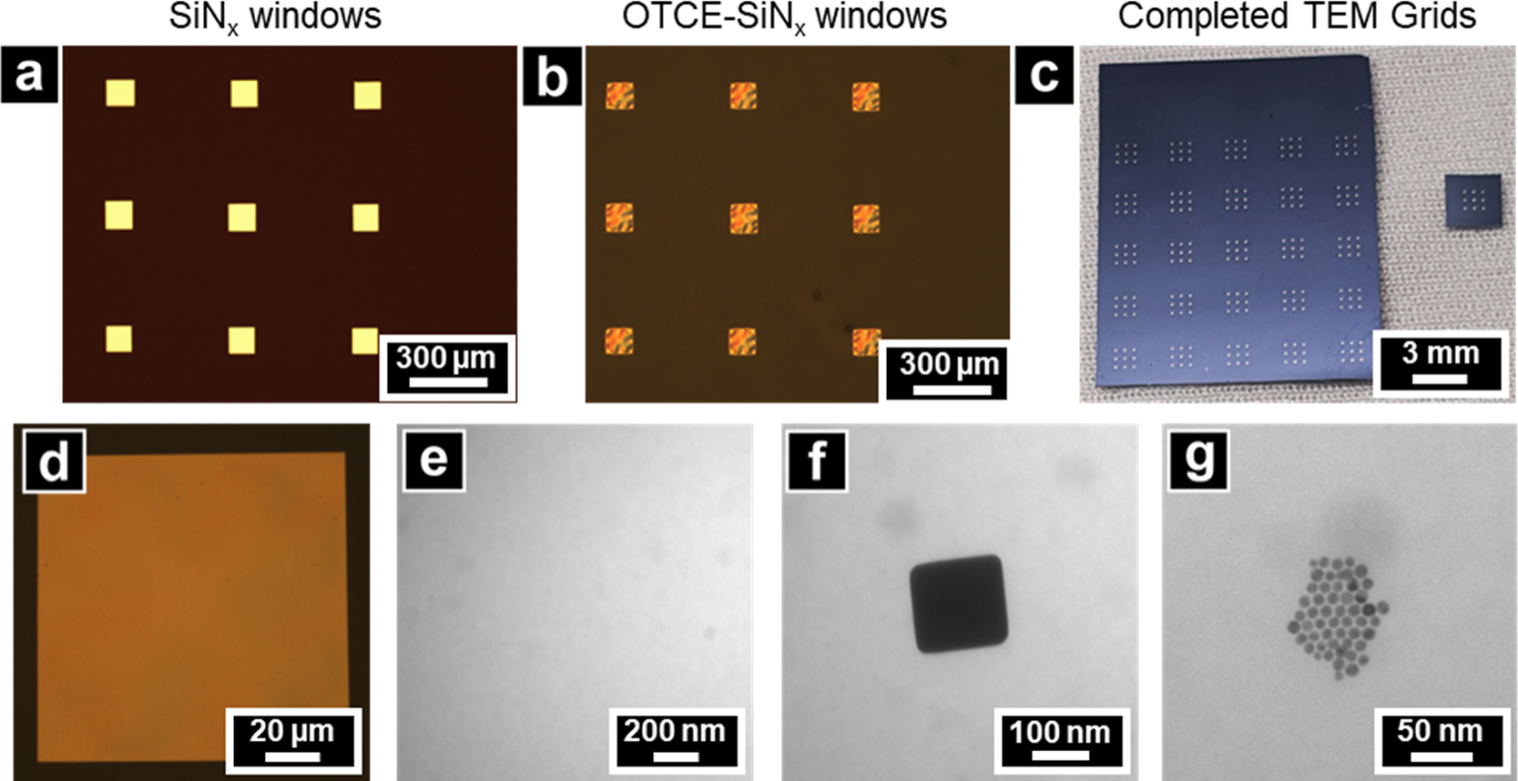
Optical images of OTCE–SiN_*x*_ windows. (a) SiN_*x*_ windows, (b) after pyrolysis forming OTCE, and (c) chip and single TEM grid level. Optical (d) and TEM (e) images of bare OTCE–SiN_*x*_. TEM micrographs of Au nanocubes (f) and Au nanospheres (g).

LM-TEM and HR-TEM imaging of nanoparticles supported on OTCE–SiN_*x*_ windows were performed. Electron micrographs in Figure 2e–g of a clean OTCE–SiN_*x*_ window, OTCE–SiN_*x*_ windows with 140 nm edge-length Au nanocubes, and 8 nm diameter Au nanospheres show clear structural identification with minimal background contribution from the OTCE–SiN_*x*_ window. HR-TEM and HAADF-STEM images of Au nanospheres supported on OTCE–SiNx windows obtained a spatial resolution of 0.14 nm (Figure 3). Au nanospheres possess a face-centered cubic crystal structure and exhibit a cyclic penta-twinned atomic arrangement. This penta-twinned configuration was verified using fast Fourier transform (FFT) patterns, which reveal five {111}-type twin boundaries arranged radially along the [110] longitudinal direction.^58^ Both HR-TEM and HAADF imaging are crucial to measure the structure of nanomaterials with the aid of complementary techniques like energy dispersive spectroscopy (EDS) and electron energy loss spectroscopy (EELS).

**Figure 3:**
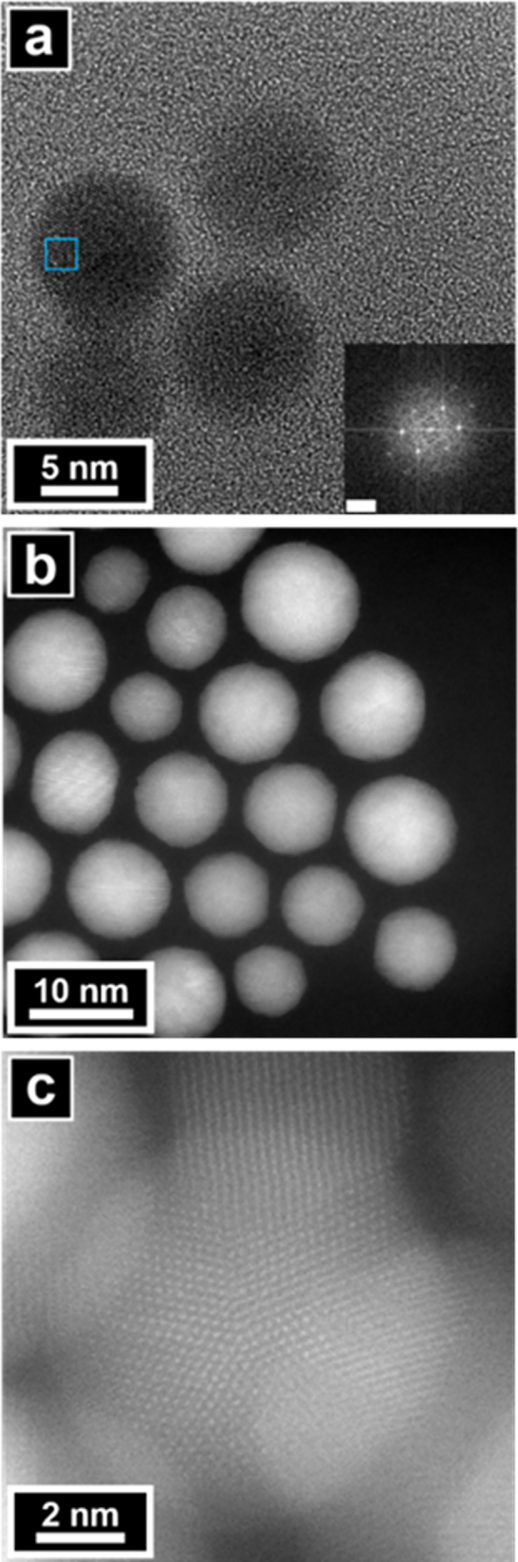
(a) HR-TEM and (b,c) HAADF-STEM micrographs of Au nanospheres. Inset in (a) is FFT of the area indicated by a blue box, and the scale bar represents 5 nm^–1^.

Previous IL-TEM studies that employ commercial carbon-coated Au TEM grids have identified instability due to delamination of the carbon layer as a possible concern.^37^ Further, carbon corrosion can occur at high anodic potential and acidic conditions^59,60^ and could be a concern for thin-film carbon electrodes. To assess the stability, an OTCE–SiNx chip was subjected to voltammetric cycling in an aqueous 0.1 M H_2_SO_4_ solution (Figure 4). The facile, outer-sphere, 1-electron reduction of ruthenium hexaammine ([Ru­(NH_3_)_6_]^3+^) was chosen to benchmark electrochemical activity.^61^ Reversible features of the [Ru­(NH_3_)_6_]^3+^ CVs remain intact after stability test CVs to 1.6 V vs Ag/AgCl (Figure 4aand c), indicating insignificant deterioration to the OTCE–SiN_*x*_ under this condition and offering a reasonable operational window for OTCE–SiN_*x*_ use. At extended windows up to 2.0 V vs Ag/AgCl (Figure 4b and c), electrochemical performance deteriorated, along with visible signs of delamination of the carbon film confirmed with optical microscopy (Supporting Information, Figure S4), to define the potential limit for electrochemical applications of the OTCE–SiN_*x*_ grids. Further corroboration of stability tests by Raman and XPS is presented in the Supporting Information, Figure S4.

**Figure 4:**
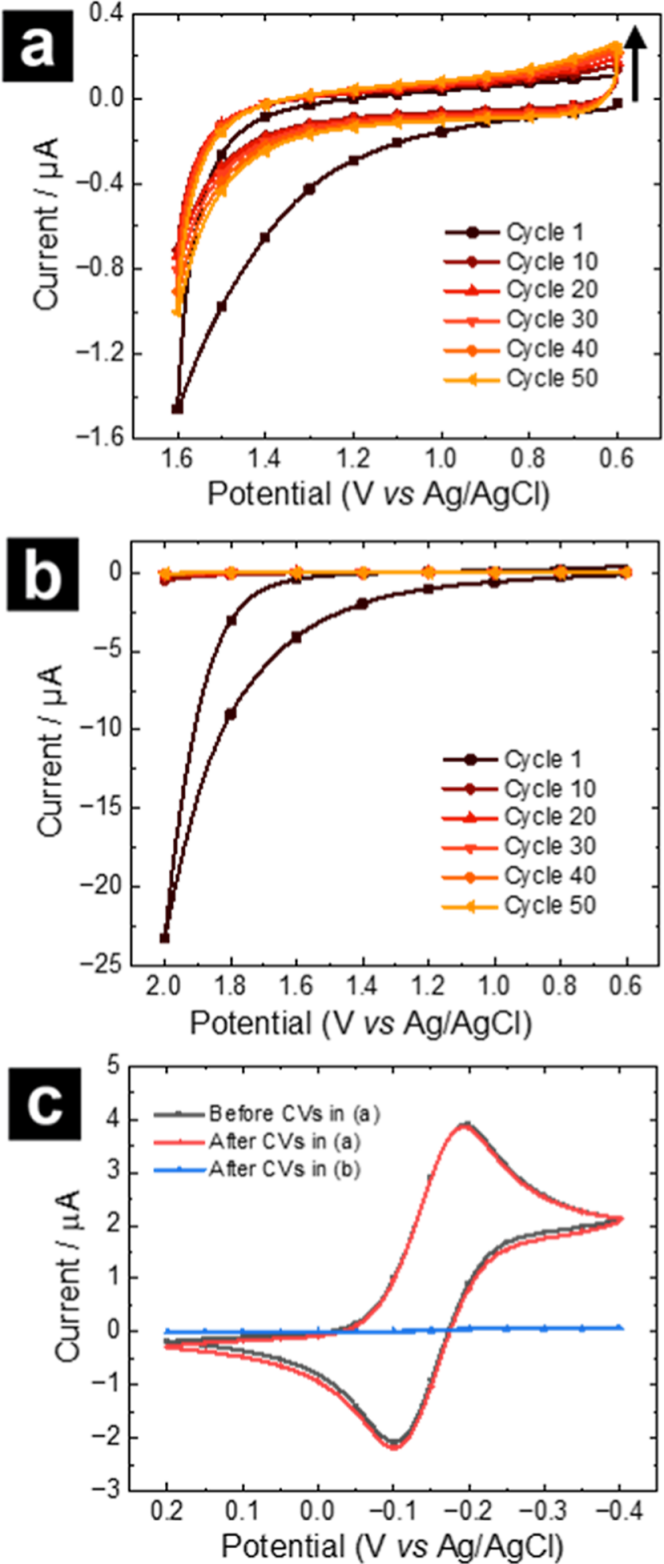
Selected cycles from CV cycling of an OTCE–SiN_*x*_ chip in 0.1 M H_2_SO_4_ at 100 mV/s for (a) 0.6 to 1.6 V and (b) 0.6 to 2.0 V potential window. (c) CV characterization of the OTCE–SiN_*x*_ chip with 5 mM [Ru­(NH_3_)_6_]­Cl_3_ in 100 mM KCl, before and after stability test CVs in (a) and (b). Scan rate for (c) is 10 mV/s. CVs are not corrected for OTCE sheet resistance. The increase in current with the CV number observed at 0.6 V in (a) is suspected to arise from the reduction of oxygen generated from the OER at 1.6 V (see Supporting Information Figure S6).

Results of nanoscale voltammetric SECCM imaging of [Ru­(NH_3_)_6_]^3+^ reduction are summarized in Figure 5. The SECCM nanopipette was positioned directly over an OTCE–SiN_*x*_ window and a CV was collected after the approach, repeated over a 7 μm^2^ area at a pixel spacing of 350 nm. The extracted limiting current (*I*_lim_) at −0.75 V vs Ag/AgCl from CVs is shown in Figure 5b. Voltammograms exhibited the expected (quasi) steady-state sigmoidal current response for mass transport encountered in SECCM (Figures 6a and S5a).^62^ Average *I*_lim_ (±standard deviation) is 6.3 ± 0.6 pA across the OTCE–SiN_*x*_ window and is approximately 10% of the magnitude of the steady-state current of an inlaid disk of the same dimension as the nanopipette opening (i.e., ca. 50 nm. See Supporting Information, Figure S5a), as expected for the SECCM configuration.^63^

**Figure 5:**
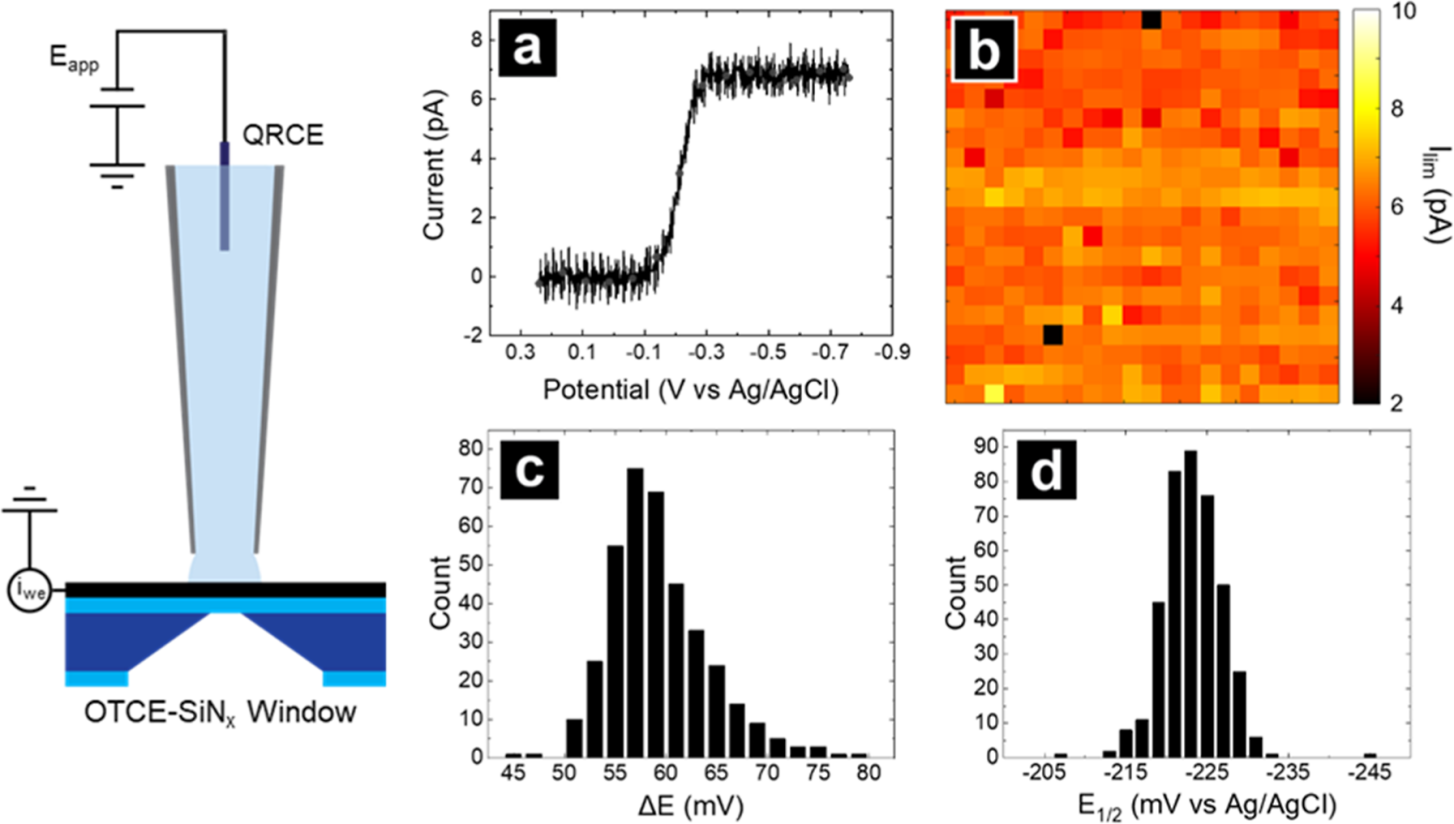
SECCM voltammetric mapping of an OTCE–SiN_*x*_ window with [Ru­(NH_3_)_6_]^3+^ reduction (left). (a) Representative SECCM voltammogram (forward sweep only) at one pixel from (b) the CV current map of the extracted limiting current at −0.75 V vs Ag/AgCl. The nanopipette (I.D. ca. 50 nm) was filled with 5 mM [Ru­(NH_3_)_6_]^3+^ in a 100 mM KCl supporting electrolyte. Voltammograms were recorded at a scan rate of 1 V/s with a pixel spacing of 350 nm for a total scan area of 7 × 7 μm. Histograms of (c) Δ*E* and (d) *E*_1/2_ analyzed from SECCM voltammograms (*N* = 400).

**Figure 6:**
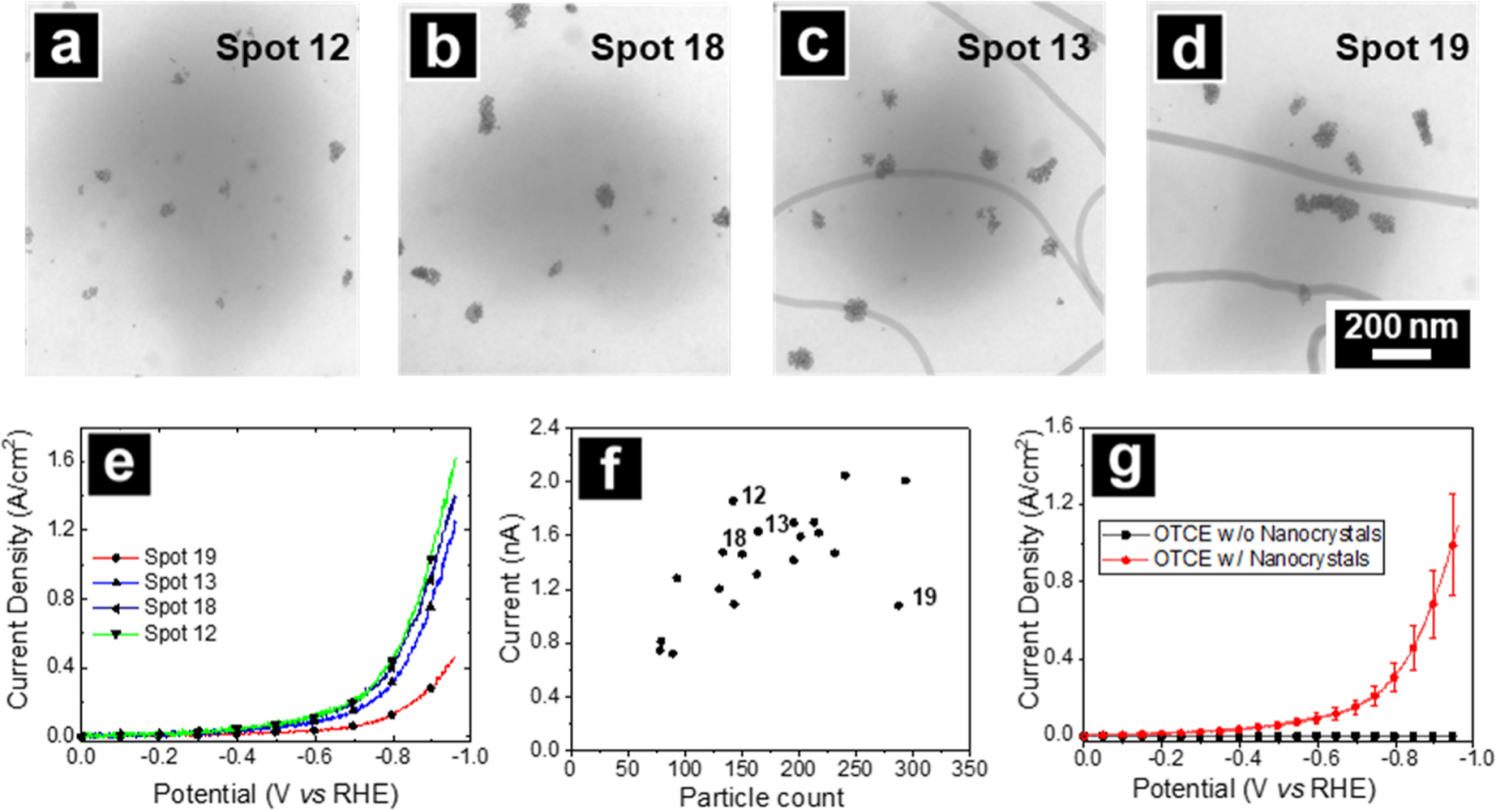
Selected LM-TEM images of locations on OTCE–SiN_*x*_ where SECCM spot LSVs were acquired for (a) spot 12, (b) spot 18, (c) spot 13, (d) and spot 19. Spots are numbered in the order the measurements were acquired. The droplet footprint is seen as a shadow in the center of each image. The nanopipette probe (I.D. ca. 800 nm) contained 0.1 M HClO_4_ and LSVs were recorded at 1 V/s. The lines present in (c) and (d) are attributed to impurity in the SiN_*x*_ which could serve as fortuitous markers for identifying specific areas and do not impact the electrochemical activity of the carbon layer. (e) Corresponding SECCM spot LSVs for the areas in (a–d) for HER at Au nanospheres supported on OTCE–SiN_*x*_. (f) Scatter plots of the background-subtracted substrate current at −0.96 V vs the RHE substrate potential for 20 independent SECCM spot measurements. Data points for selected spots in (a–e) are indicated in (f) with their index number labeled to the left. (g) Average LSVs for 20 SECCM spot measurements on areas with nanocrystals (red) compared to areas without nanocrystals (black). Error bars are ± 1 SD for the corresponding LSV. All voltammograms are presented in the Polarographic or Texas convention.

Analyses of kinetic parameters calculated from each pixel are presented in Figure 5c and d. The distribution of peak splitting (Δ*E* = |*E*_3/4_ – *E*_1/4_|) is centered at 57 mV (Figure 5c), indicative of a 1 electron transfer reaction and meets the Tomes criterion of reversibility.^64^ The half-wave potential, *E*_1/2_, yielded a distribution of −0.223 ± 0.020 V vs Ag/AgCl (Figures 5d and S5b), in agreement with the formal potential of ruthenium hexaammine reduction measured at platinum^65^ and carbon substrates.^66^ The narrow distribution in electrochemical figures of merit (limiting current, *E*_1/2,_ and Δ*E*) indicates a uniform electrode response with ideal electron transfer properties.^67^ Notably, we encountered no instances of pipettes crashing through the OTCE windows. Altogether, this analysis supports that OTCE–SiN_*x*_ windows prove ideally suitable substrates for SECCM, single-entity electrochemistry (SEE), or other nanoscale electrochemical interrogation.

Lastly, we demonstrate the application of OTCE–SiN_*x*_ windows as support electrodes for correlative SECCM–TEM studies for HER on ∼8 nm Au nanosphere catalysts. Drop casting nanocrystals onto OTCE–SiN_*x*_ windows resulted primarily in clusters comprising a few to 10s of nanocrystals. A relatively large nanopipette probe (∼800 nm diameter) was used to acquire electrochemical measurements of these small nanocrystal ensembles. Ensuring multiple nanoparticles per SECCM meniscus contact mitigated difficulties in measuring the low electrocatalytic current associated with single 8 nm nanocrystals which could be ≤ 2 pA and comparable to the peak-to-peak noise levels. The nanopipet was filled with 0.1 M HClO_4_ and linear sweep voltammograms (LSVs) of HER were acquired at multiple locations across the sample. To facilitate co-location, SECCM footprints were located first by SEM and then by LM-TEM.

Results of 20 independent voltammetric measurements are summarized in Figure 6. Footprints observed in LM-TEM images did not obscure nanosphere imaging and enabled identification of particles within the measurement area for each LSV (Figure 6a–d). Background LSVs were acquired from locations where no particles were present. Background-subtracted LSVs were normalized to the estimated particle surface area from TEM image analysis (Figure 6e, see Supporting Information for details). Figure 6f shows that background-subtracted LSV currents at *V* = −0.96 V vs RHE scale with the total number of particles within the wetted area, as expected for an increase in catalyst loading. Average LSVs are presented in Figure 6g. Variations of HER activity were noted, along with additional limitations of the geometric catalytic activity compared to the electrochemical surface area described in the Supporting Information. For data collected here, the distribution of the nanospheres, that is, cluster size and count differences, does not depict a clear relationship with the observed current density (Supporting Information).

Further studies at the intermediate scale between single nanocrystal electrocatalysis and macroscale studies, on a few particles in different arrangements, could provide key insights into catalyst operation and help bridge the gap between SEE measurements versus macro-electrodes or state-of-the-art reactors.^68,69^ Overall, this result represents an SECCM measurement on clusters of sub-10 nm nanoparticles and demonstrates the utility of the prepared OTCE–SiN_*x*_ for future correlative SECCM–TEM studies.

## Conclusions

In this report, we described the fabrication of carbon-coated, SiN_*x*_ windows suitable for correlative analysis by both TEM and SECCM. By employing OTCE as the electrode material on a 60 nm SiN_*x*_ window support, the required transparency and dimension (<100 nm thick) for LM and HR-TEM are achieved. Images of metal nanoparticles with TEM and HAADF-STEM imaging were collected and the atomic structure of Au nanospheres was well resolved. Electrochemical mapping at OTCE–SiN_*x*_ windows of the reduction of ruthenium hexaammine showed spatially homogeneous electrochemical activity from the evaluation of the limiting current, *E*_1/2_, and Δ*E*. The SECCM of clusters of Au nanosphere particles supported on an OTCE–SiN_*x*_ window demonstrated the possibility of accessing single-entity measurements on smaller particles in the future.This data demonstrates that OTCE−SiN_*x*_ windows are a robust, versatile platform for correlative nanoscale structure and electrochemical analysis by SECCM and can enable the advancement of structure–activity SEE studies with nanomaterials.

## Supplementary Material



## Data Availability

Data reported used to generate results and other findings of this study are available from the corresponding author upon reasonable request.

## Notes

Additional optical images of OTCE–SiN_*x*_; SEM micrographs of nanopipette probes; additional plots for nanoscale characterization with SECCM; analysis of colocated TEM micrographs; analysis of SECCM spot voltammetric measurements; particle cluster distribution; and supporting information references and figures (PDF)
