# Supplementary material for: Optically Transparent Carbon–Silicon Nitride Windows for Correlative Structural and Electrochemical Analysis of Nanomaterials
Source: Anal Chem. 2025 May 23;97(22):11798–805. doi: 10.1021/acs.analchem.5c01403 (PMC12163881; doi:10.1021/acs.analchem.5c01403)
Supplement: Supplementary file 1 [file ac5c01403_si_001.pdf]

Supporting information

Optically Transparent Carbon-Silicon Nitride Windows for Correlative Structural and Electrochemical Analysis of Nanomaterials

Sasha E. Alden,[1] Oluwasegun J. Wahab[1], Lingjie Zhang,[1] Kelly L. Vernon,[1] Baixu Zhu,[2] Kathleen O. Bailey,[1] Xingchen Ye,[2] and Lane A. Baker[1]*

[1]Department of Chemistry, Texas A&M University, College Station, Texas 77843
[2]Department of Chemistry, Indiana University, Bloomington, Indiana, 47405

Table of Contents

Chemicals and Nanoparticle Synthesis	S1
Additional images and surface characterizations of OTCE-SiN_x	S2
SEM micrographs of nanopipette probes	S3
SECCM Instrumentation and Additional plots for electrochemical characterization	S4
Analysis of co-located TEM micrographs	S5
Analysis of SECCM spot voltammetric measurements	S6
Particle cluster distribution	S7

Section S1. *Chemicals and Nanoparticle Synthesis*

Aqueous solutions were prepared with deionized water (resistivity = 18.2 MΩ•cm, Thermo Scientific). The following chemicals were used as received: propylene glycol monomethyl ether acetate ($CH_3CO_2CH(CH_3)CH_2OCH_3$, Sigma-Aldrich), potassium chloride (KCl, VWR), methanol (CH_3OH, 200 proof, Fisher Chemical), potassium hydroxide pellets (KOH, Fischer Chemical), acetonitrile (CH_3CN, Fisher Chemical) and ruthenium hexammine chloride ($(Ru(NH_3)_6)Cl_3$, 98%, Aldrich). Tetrachloroauric acid trihydrate ($HAuCl_4 \cdot 3H_2O$, 99%, Sigma-Aldrich), borane tert-butylamine (TBAB, 97%, Sigma-Aldrich), oleylamine (50%, TCI America) and toluene (anhydrous, 99.8%, Sigma-Aldrich).

Au nanocubes were synthesized as previously reported by Choi et. al.[1] using a seed mediated-growth method. Synthesis yielded cubes with an edge length of ~140 nm capped with hexadecyltrimethylammonium bromide (CTAB) ligands. Nanocubes were dissolved in water. Au nanospheres were synthesized by a previously reported method of Jeong et. al.,[2] where 10 ml of oleylamine was loaded into a 50 mL three-neck- flask. The oleylamine was evacuated for 30 min. After N_2 refilling, 10 mL of anhydrous toluene and 0.25 mmol of $HAuCl_4 \cdot 3H_2O$ were swiftly added into the reaction system. After cooling the reaction system to 15 °C by an ice bath, a solution consisting of 0.25 mmol (22 mg) of TBAB, 1 mL of oleylamine and 1 mL of anhydrous toluene was quickly injected into the reaction mixture. The reaction was stirred for 1 hour at 15 °C. Au nanospheres were precipitated by acetone and collected by centrifugation. Final products were redispersed in anhydrous toluene.

Section S2. Additional images and surface characterizations of OTCE-SiN_x

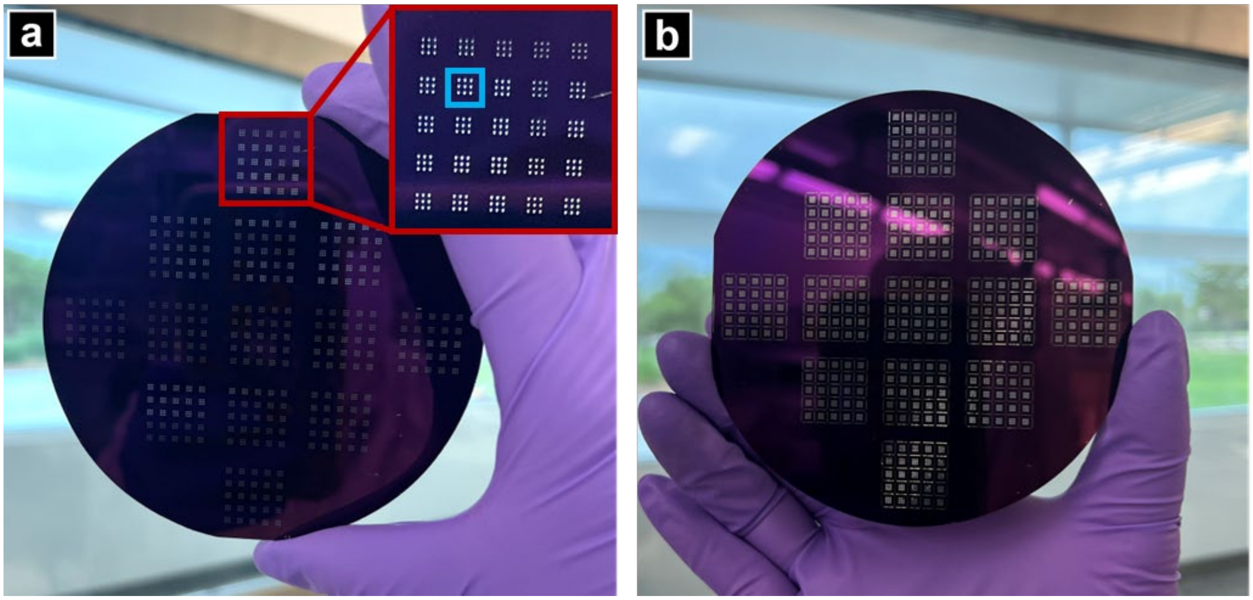

Figure S1. Photographs of OTCE-SiN_x TEM grids at the wafer level of (a) the window and (b) reverse side, respectively. Inset in (a) highlights the chip (red box) and single TEM grid (blue box) level which can be easily broken out of the wafer due to the scoring lines partially etched on the reverse side of the wafer (b). Wafer size: 4-inch diameter.

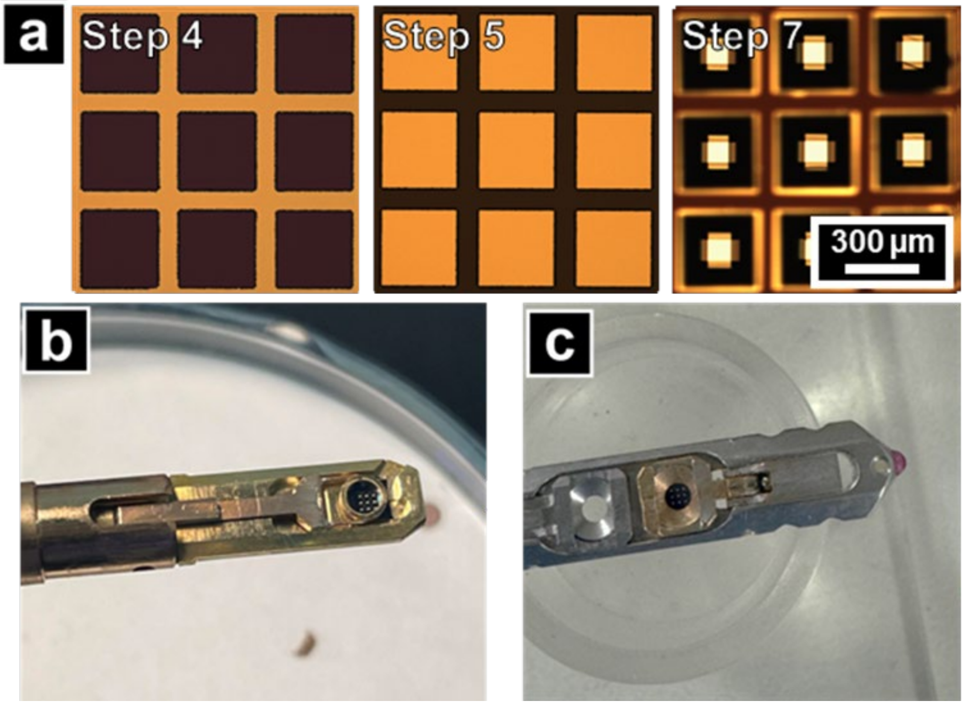

Figure S2. (a) Optical images of the reverse side of OTCE-SiN_x grids at steps of microfabrication as indicated in Figure 1 of the photolithography defined pattern (step 4), RIE etch of SiN_x (step 5), and SiN_x windows after KOH Si etch. Pictures of the OTCE-SiNx grid in (b) Titan Themis 300 S/TEM, and (c) JEOL 1200 EX TEM

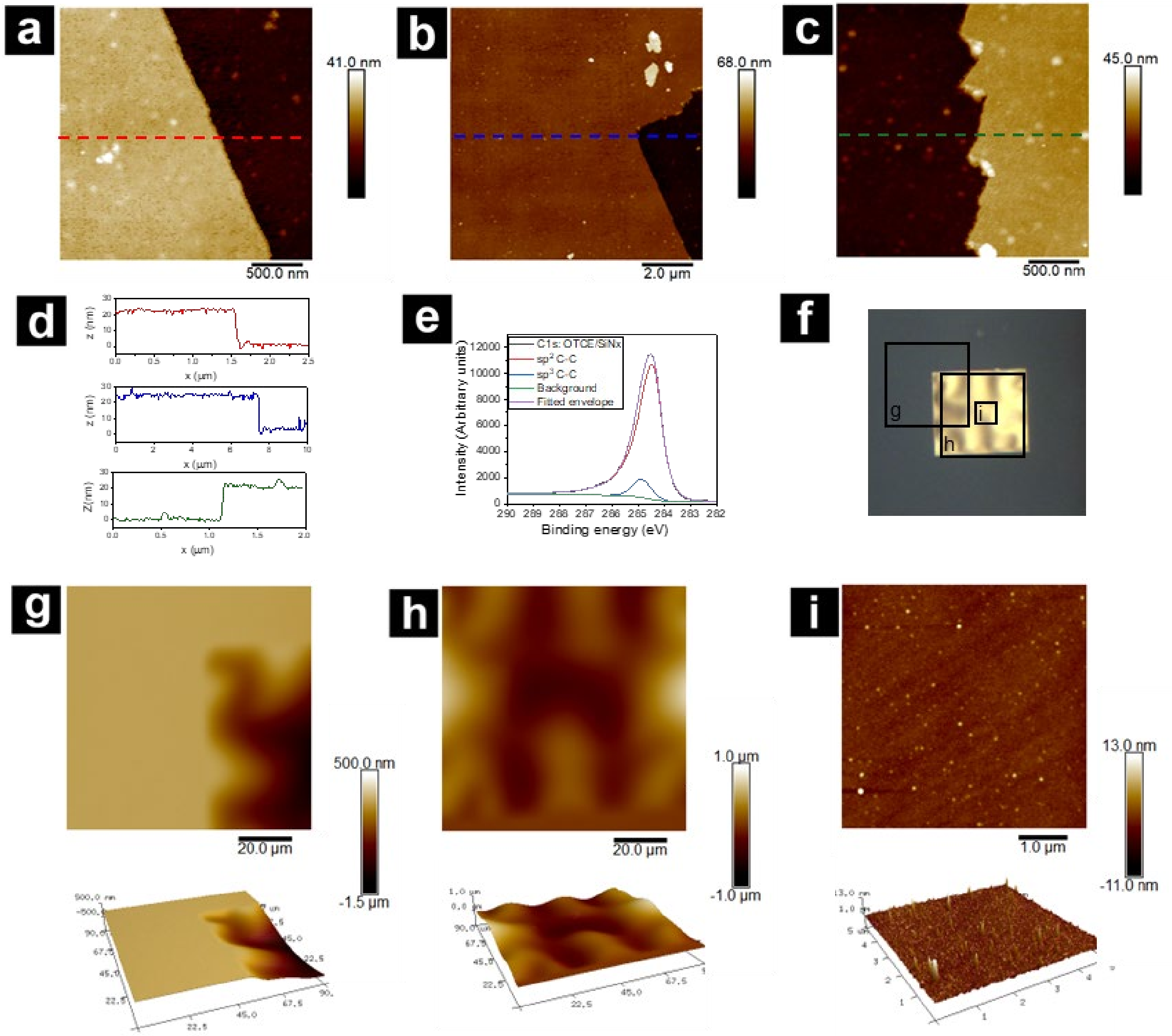

Figure S3. (a-c) AFM topography images of scratched OTCE-SiN_x, revealing the OTCE surface and the underlying SiN_x-Si support. (d) Cross-sectional line profiles extracted from the AFM images (a-c) at the red, blue, and green dashed lines, demonstrated a film thickness of approximately 22 nm in all three cases. (e) Fitted C 1s XPS spectra of OTCE-SiNx. Showing 91.89 % and 8.11 % sp^2 and sp^3 C-C functionality respectively. (f) Optical microscope image of OTCE-SiN_x, with boxes indicating the locations of AFM images (g-i). AFM topography images presented in both 2D and 3D: (g) covering both the on-window and off-window regions, (h) centered on the window, and (i) zoomed into a small area within the window. The root-mean-square (rms) surface roughness of the carbon film-covered areas a, b, and c is 1.3 nm, 1.5 nm, and 1.1 nm, respectively, on SiNx|Si-supported regions. Additionally, based on the topography image (g), the rms surface roughness increases from 1.51 nm in Si frame-supported areas to 175 nm on the frame itself. The rms surface roughness over the entire AFM image in (h) is 202 nm. This indicates that the primary structure of the carbon film is flat, conforming to the underlying support material. Specifically, the film remains flat on SiNx|Si while becoming rougher on the SiNx window. The highest roughness of 202 nm rms observed on the SiN_x window area is comparable to an electrode polished with 0.2 µm polishing suspension. AFM was carried out with a Dimension Icon AFM (Bruker).

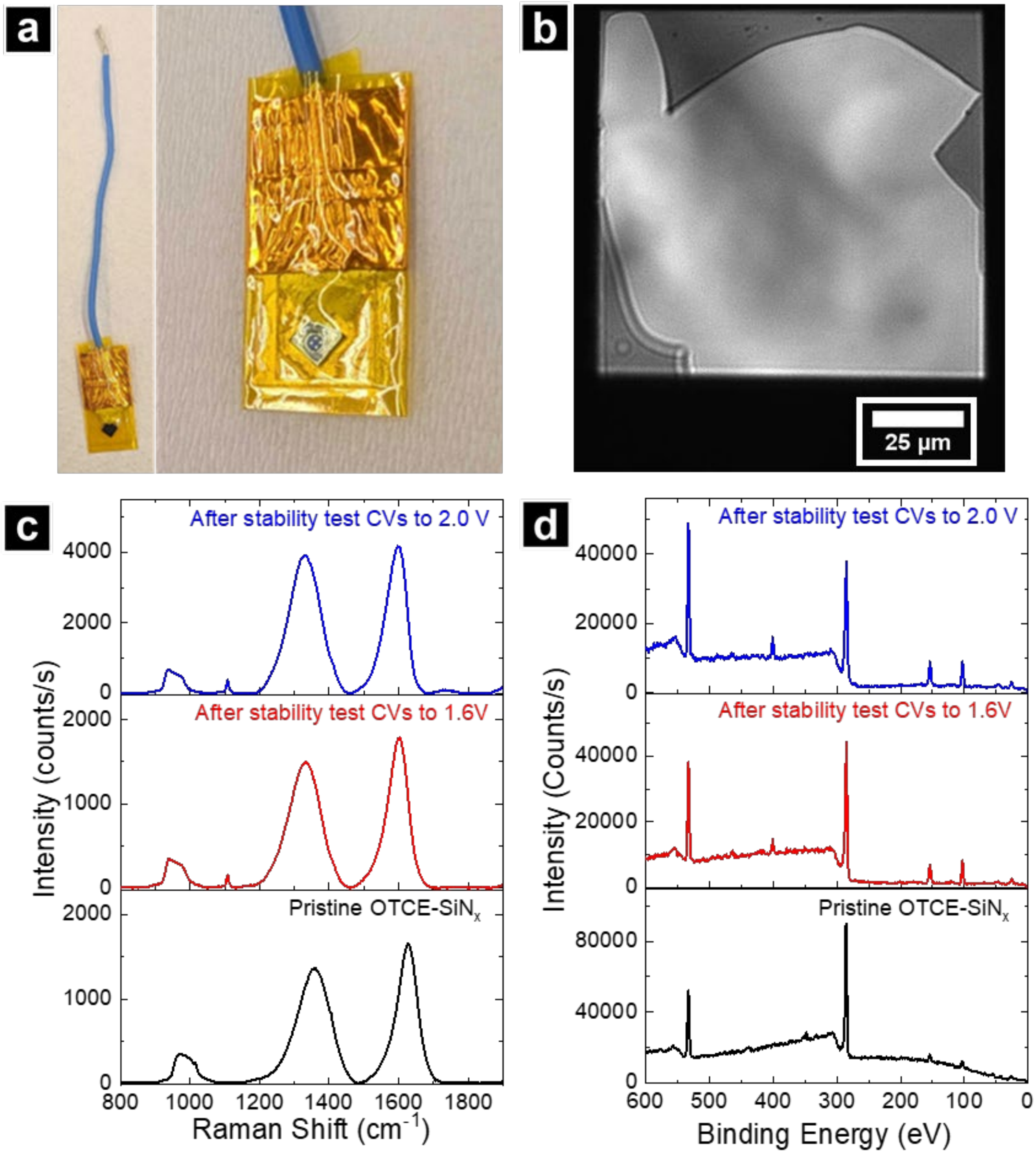

Figure S4. (a) Photograph of Kapton-masked OTCE-SiN_x for macroscale CVs. **(b)** Optical image of delaminated OTCE-SiN_x windows after CV cycles in 0.1 M H_2SO_4 to 2.0 V *vs* Ag/AgCl (see Figure 4 of the main manuscript). **(c)** Raman spectra acquired on OTCE SiNx grid before and after stability test CVs. The ratio of the intensity of the D to G bands at 1335 cm^{-1} and 1600 cm^{-1} respectively, increased from approximately 0.8 to 0.95 after CVs in 0.1 M H_2SO_4 to 2.0 V *vs* Ag/AgCl **(d)** XPS spectra were collected on OTCE SiNx grids before and after stability test CVs. The intensity of the C1s peak at 285 eV substantially decreased after stability test CV scanned to 2.0 V, relative to the Si2p, Si2s, N1s, and O1s peaks at 103 eV, 155 eV, 401 eV, and 533 eV, respectively. Raman spectra were acquired on Horiba Jobin-Yvon LabRam HR spectrometer, and XPS spectra were acquired on SPECS EnviroESCA.

Section S3. SEM micrographs of nanopipette probes

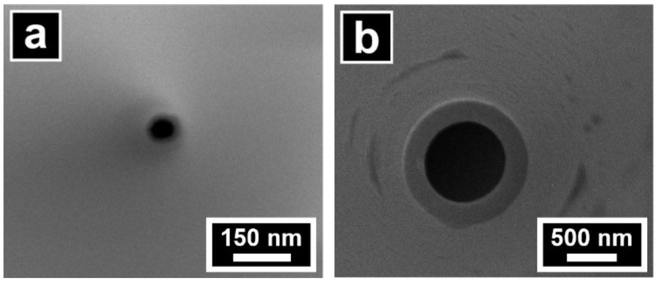

Figure S5. Electron micrographs of pipette tips with ca. (a) 50 and (b) 800 nm I.D. Pulling parameters were as follows for (a) HEAT = 60, FIL = 3, VEL= 45, DEL = 180, PULL= 155 and (b) HEAT = 500, PULL= 0, VEL = 40, TIME = 175, and PRESSURE = 500. Nanopipettes are sputter coated with Au-Pd before SEM imaging. SEM images were acquired on QUANTA field emission SEM, with an acceleration voltage of 20 kV.

Section S4. SECCM Instrumentation and additional plots for electrochemical characterization

A AgCl-coated Ag quasi-reference counter electrode (QRCE) wire was back-inserted into an electrolyte solution filled nanopipette. Coarse control of the tip utilized a manual x,y,z stage and a stepper motor (M-111.1DS, Physik Instrumente) with controller (C.863, Physik Instrumente). Fine control was realized by x, y, and z piezo actuators (Nano-MET20, Mad City Labs), with 20 µm total travel distance. Coarse control of the sample utilized a manual microscope mounting adapter (Mad City Labs), and fine controlled with a XY-scanner (Nano-BioS200, Mad City Labs) with 200 µm total travel distance in each direction. Piezoelectric positioners were controlled through a CompactRIO Systems FGPA (NI-9147, NI-9220, NI-9264, National Instruments) from a PC running custom LabVIEW code (LabVIEW 2024, National Instruments). A custom-built headstage (voltage follower) was used to apply voltages (E_{app}) at the QRCE for electrochemical measurements and position feedback. Current at the working electrode (i_{we}) was amplified by a patch-clamp amplifier (Axon Axopatch 200B, Molecular Devices) connected to the same headstage, and the signal was collected by the same CompactRIO Systems mentioned above. The instrument was operated in a Faraday cage, and the sample of interest was mounted in a humidified chamber. During the experiment, the current signal and the location of the piezoelectric positioners were recorded simultaneously.

A voltammetric hopping mode scanning protocol was utilized for electrochemical mapping. A bias of -0.75 V vs Ag/AgCl was applied during the nanopipette approach (2 µm/s) to trigger a current response from meniscus contact with the working electrode (OTCE-SiN_x window), halting the approach. A waveform was then applied to collect a cyclic voltammogram (CV), or linear sweep voltammogram (LSV), and the current response measured. After electrochemical measurement, the nanopipette was retracted 10 µm (10 µm/s) and moved to the next pixel. This program continued in a raster scan across the substrate, generating a CV or LSV at every pixel.

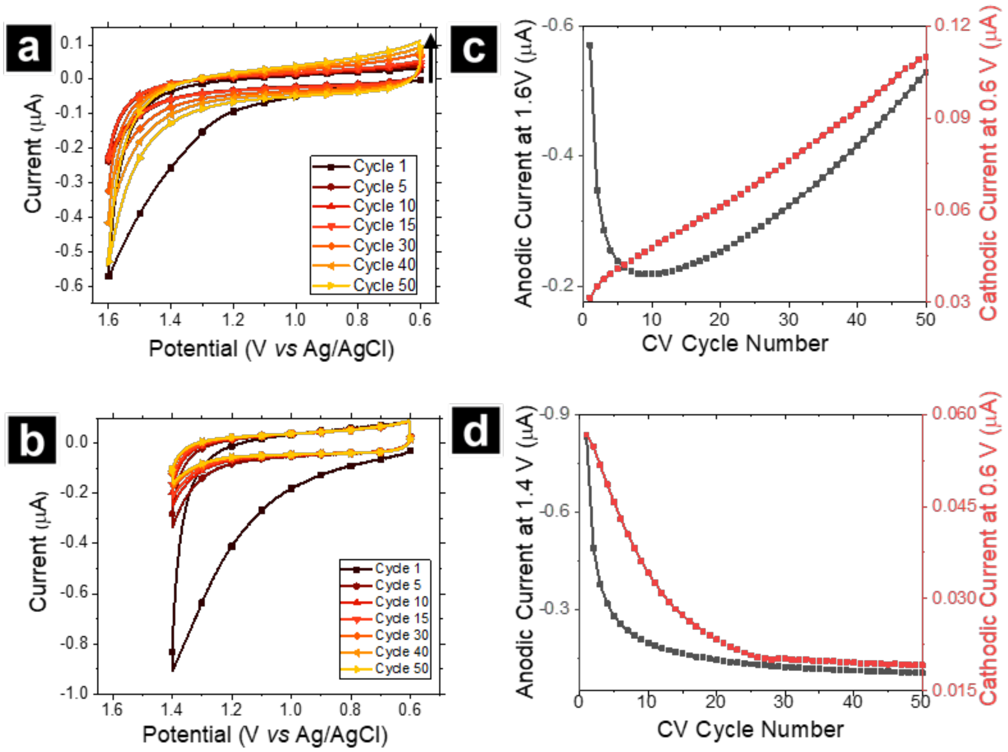

Figure S6. Selected cycles from CV cycling of an OTCE-SiN_x chip in 0.1 M H_2SO_4 at 100 mV/s for (a) 0.6 V to 1.6 V; and (b) 0.6 V to 1.4 V potential window. (c) A plot of cathodic current at 0.6 V and anodic current at 1.6 V for CVs presented in (a). (d) A plot of cathodic current at 0.6 V and anodic current at 1.4 V for CVs presented in (b). This result shows that when the CVs are limited to 1.4 V vs Ag/AgCl, the oxidation current at 1.4 V decreases after a few cycles, consistent with carbon cleaning at the electrode surface. The remaining cycles exhibited a consistent current response over 50 cycles with no signs of instability. However, for CVs extended to 1.6 V, a reduction wave at 0.6 V that increases with cycle number is observed. This wave at 0.6 V is suspected to arise from the reduction of oxygen generated from the OER at 1.6 V, which also tends to increase after a few CV cycles.

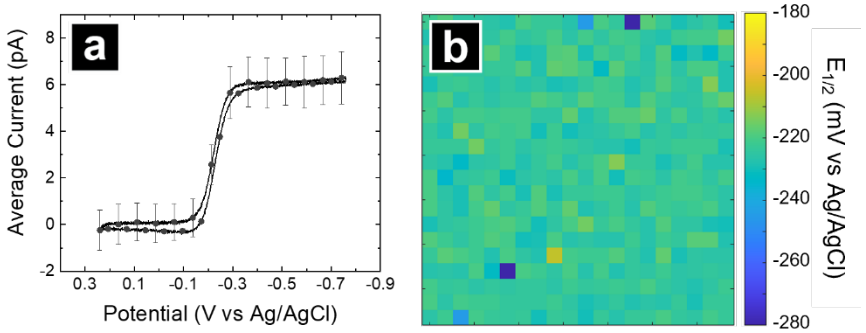

Figure S7. Additional analysis of SECCM data presented in Figure 6. (a) Averaged SECCM CV from current map, error bars represent standard deviation. (b) Color map of $E_{1/2}$.

Section S5. Analysis of co-located TEM micrographs

TEM images for SECCM spot measurements were analyzed to extract the total number of particles within the footprint of the electrochemical droplet cell. While particles can be counted manually, image analysis with ImageJ[3] and MATLAB (version R2023b, MathWorks, Inc.) was used to ensure a more consistent analysis across multiple spot measurements. Background subtraction using the sliding paraboloid function with a rolling ball radius of 50 to 100 pixels was employed to remove electrolyte footprint marks, resulting in a clear background. Contrast and brightness adjustments were made, and images were converted to binary format for MATLAB processing. Titles at the base of the TEM images were cropped off, and objects with pixel dimensions less than 2 nm were excluded. The 'Segmentation application' in MATLAB was then used to identify clusters and nanoparticles.

Properties of the features and objects extracted with MATLAB were analyzed, and particle counts were based on fitting the dimensions of clusters to arrangements of 7-10 nm-sized disks. The output counts were significantly consistent with manual counts / visual inspection for clearly separated particles and allowed for relatively more accurate counting in clusters where manual counts became less reliable. The droplet areas were determined based on the footprint from the original TEM images, guiding which particles to include or exclude. Example TEM images before and after the processing steps are presented in Figure S8. In cases where features from the underlying SiN_x precluded reliable feature analysis (see Figure S9), careful manual counting was used. TEM images with excessive electrolyte residue within the droplet footprint (Figure S10) were excluded from consideration.

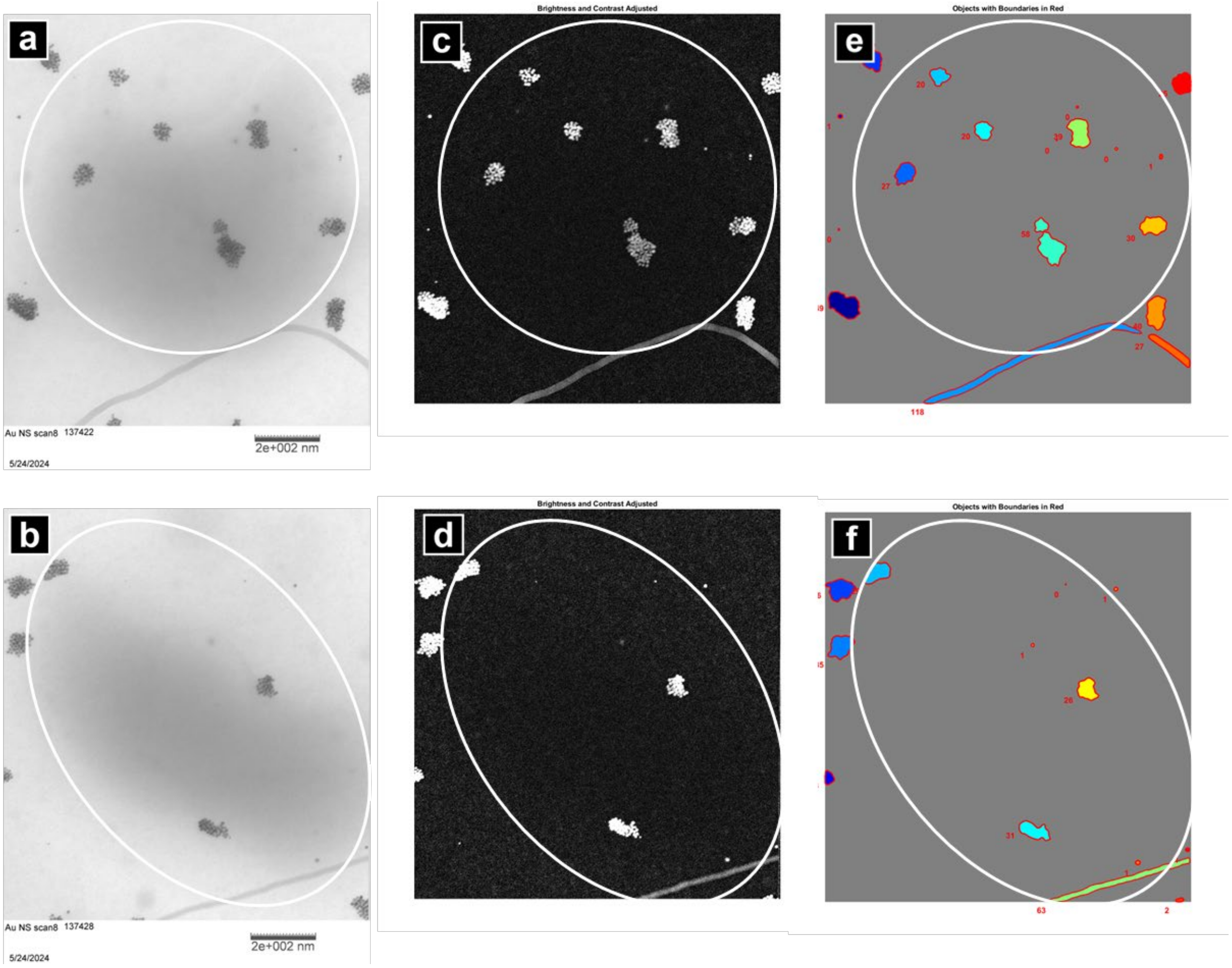

Figure S8. (a & b) Examples of TEM micrographs for SECCM spots showing the electrolyte footprint mark area where LSV was collected. (c & d) The corresponding images after optimization with ImageJ and MATLAB image processing to remove the footprint shadow and convert to binary images. (e & f) The final output of MATLAB image analysis, which includes particle counts based on identified clusters and circular particles with diameters of 7-10 nm. This was combined with visual inspection to reach the final count used to normalize electrochemical data.

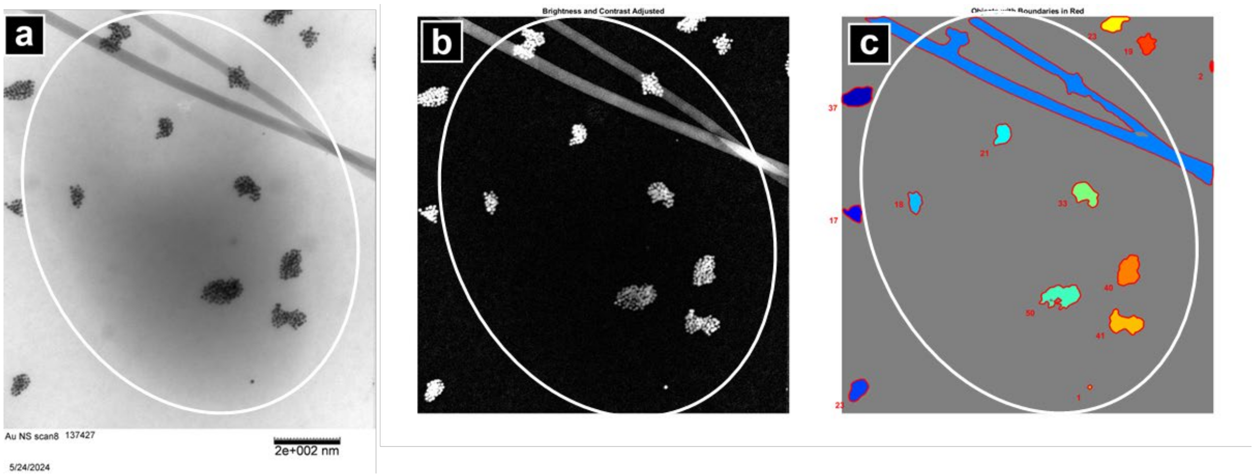

Figure S9. TEM micrographs for SECCM spots (a) with corresponding processed images before (b) and after (c) counting. The final count considered to normalize LSV currents excludes convolutions with background SiN_x morphology and resorts to manual count.

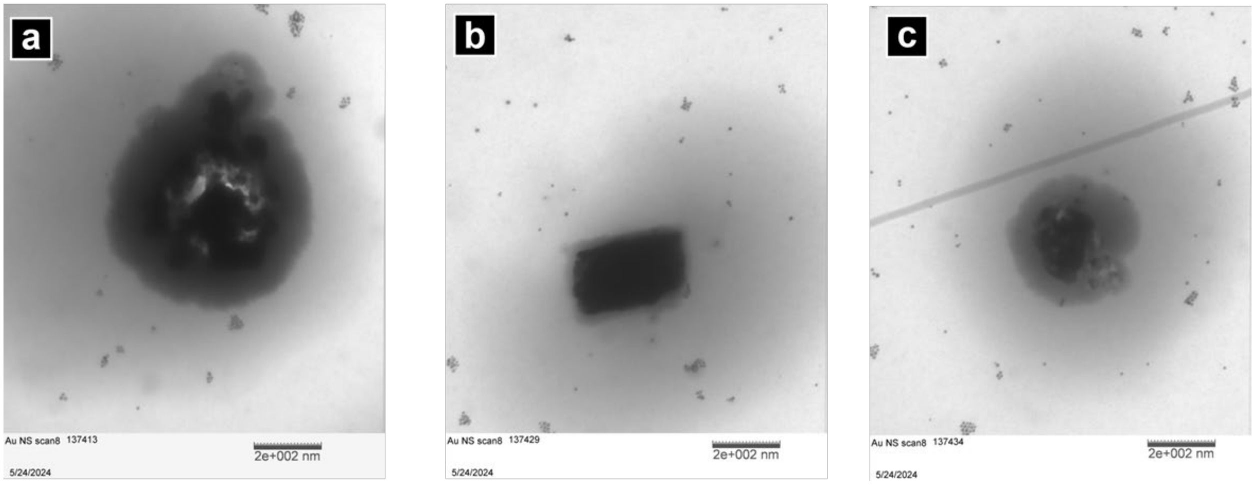

Figure S10. Examples of TEM micrographs excluded from analysis due to excessive electrolyte residue build-up within the SECCM spot.

Section S6. Analysis of SECCM spot voltammetric measurements

As described in the main text, LSVs were acquired at several sites of drop-cast samples of nanoparticles on the OTCE-SiN_x window. The current at -0.96 V vs. RHE ranged from 0.74 nA to approximately 2.0 nA. Conversely, for background LSVs acquired on blank OTCE-SiN_x areas without nanoparticles, current at the same potential ranged from 0.32 nA to 0.5 nA. (Figure S11a). The mean ± SD of the wetting diameter of 1.20 ± 0.21 μm for 25 spot measurements on blank OTCE compares well to 1.77 ± 0.14 μm diameter observed for 20 measurements taken on areas with nanocrystals. Hence, the mean of LSVs acquired from blank OTCE (Figure S11b) was used for background subtraction from spot measurements to account for background HER contributions of the support electrode (Figure S12).

Due to the background noise level relative to the catalytic activity of supported nanoparticles, potentials where the background LSV has approximately zero current (e.g., V < -0.5 V vs. RHE) could not be used to estimate the catalytic activity of the nanoparticles at this scale. Background-subtracted LSVs were then normalized to the estimated surface area based on particle counting described in Section S4, assuming the entire surface area of the approximately 8 nm nanoparticles is recruited for HER. Although this may not be the case for clusters with connected sides, this approach minimizes over-estimation of catalytic activity and provides a lower-bound estimate of catalytic activity. We consider this method more reliable than normalizing the measured LSV current to the geometric surface area of the SECCM footprint, which does not account for different catalyst loading/particle density across the spots. In future works, the SECCM protocol can be modified to estimate the electrochemical surface area (ECSA) for each spot,[3-5] and the electrocatalytic current can be normalized to the ECSA for reliable comparison to geometric current density, and for consideration of 3D particle arrangements.

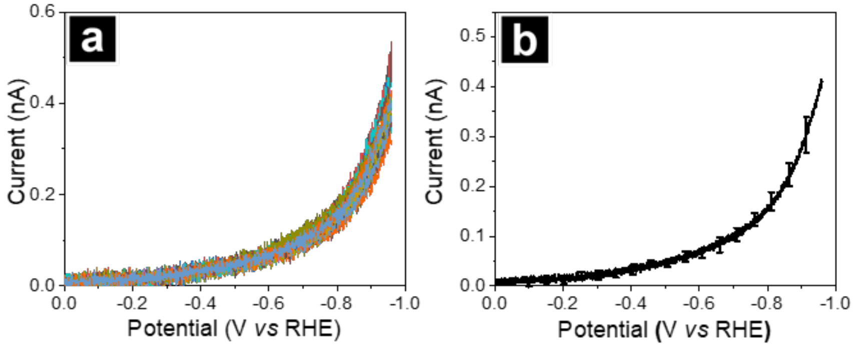

Figure S11. (a) LSVs acquired from 25 spot measurements on blank OTCE areas. (b) Plot of the mean ± 1SD of the 25 background LSVs in a.

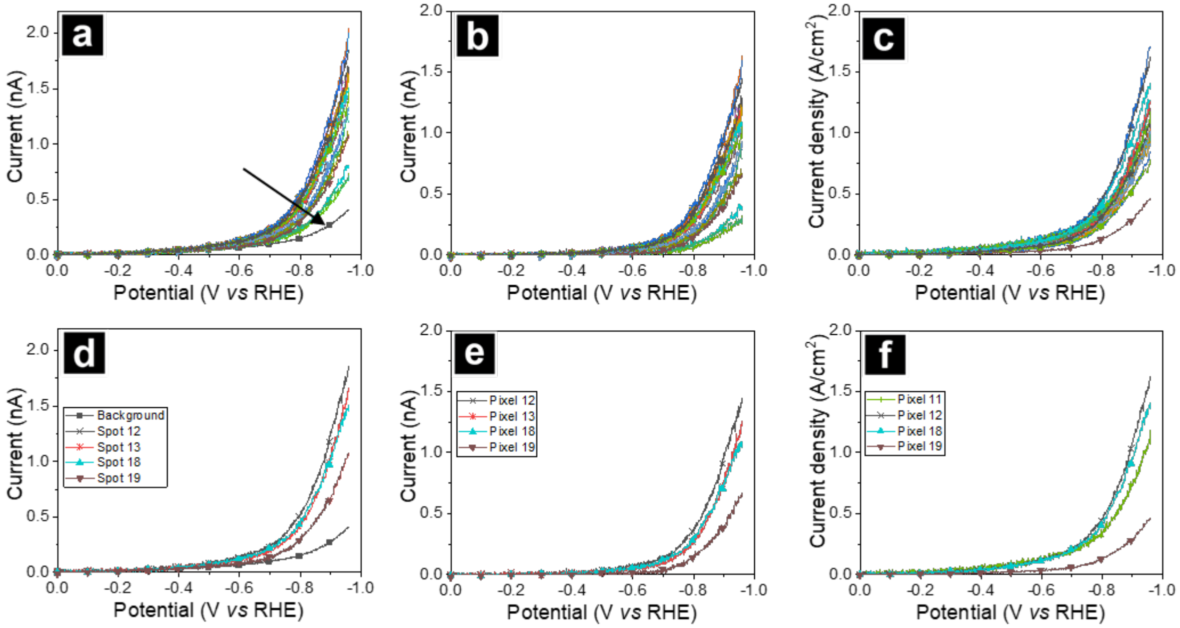

Figure S12. Details of the electrochemical data analysis. (a) Raw current LSVs from 20 SECCM spot measurements. The mean background LSV of 25 measurements is indicated with an arrow. (b) LSVs from spot measurements after subtracting background CVs (c) LSVs normalized to current density in A / cm^2, based on estimated nanoparticle area. The same plot as (a-c), are presented in (d-f) respectively to show only LSVs for spots 12, 13, 18, and 19, as presented in Figure 7 of the main text.

Section S7. Particle cluster distribution

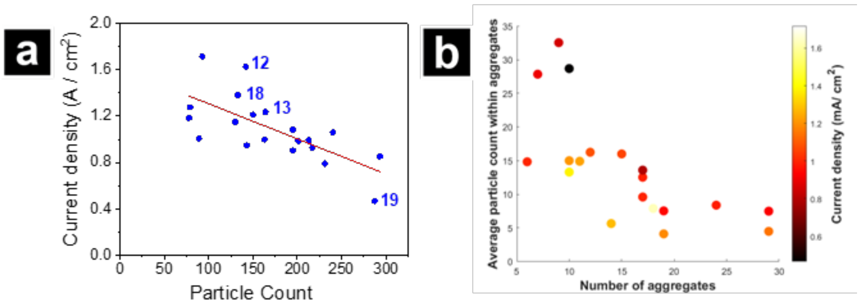

Figure S13. (a) Scatter plots of current density at -0.96 V vs RHE substrate potential for 20 independent SECCM spot measurements. Datapoints for selected spots in Figure 7 of the main manuscript are indicated in (a & b) with their index number labeled to the right. Linear fit plotted as a solid red line has an imperfect R-square (coefficient of determination) value of 0.47862. (b) Heat map for the plot of average cluster size vs number clusters. The colorscale shows the current density (A/cm^2) where LSV current at V = -0.96 V vs RHE has been normalized to particle surface area after background subtraction.

SI References

(1) Choi, M.; Siepser, N. P.; Jeong, S.; Wang, Y.; Jagdale, G.; Ye, X.; Baker, L. A. Probing Single-Particle Electrocatalytic Activity at Facet-Controlled Gold Nanocrystals. *Nano Letters* **2020**, *20* (2), 1233-1239. DOI: 10.1021/acs.nanolett.9b04640.

(2) Jeong, S.; Liu, Y.; Zhong, Y.; Zhan, X.; Li, Y.; Wang, Y.; Cha, P. M.; Chen, J.; Ye, X. Heterometallic Seed-Mediated Growth of Monodisperse Colloidal Copper Nanorods with Widely Tunable Plasmonic Resonances. *Nano Letters* **2020**, *20* (10), 7263-7271. DOI: 10.1021/acs.nanolett.0c02648.

(3) Tetteh, E. B.; Kim, M.; Savan, A.; Ludwig, A.; Chung, T. D.; Schuhmann, W. Reassessing the intrinsic hydrogen evolution reaction activity of platinum using scanning electrochemical cell microscopy. *Cell Reports Physical Science* **2023**, *4* (12), 101680. DOI: 10.1016/j.xcrp.2023.101680.

(4) Bentley, C. L.; Agoston, R.; Tao, B.; Walker, M.; Xu, X.; O'Mullane, A. P.; Unwin, P. R. Correlating the Local Electrocatalytic Activity of Amorphous Molybdenum Sulfide Thin Films with Microscopic Composition, Structure, and Porosity. *ACS Applied Materials & Interfaces* **2020**, *12* (39), 44307-44316. DOI: 10.1021/acsami.0c11759.

(5) Harris-Lee, T. R.; Turvey, T.; Jayamaha, G.; Kang, M.; Marken, F.; Johnson, A. L.; Zhang, J.; Bentley, C. L. Optimizing Amorphous Molybdenum Sulfide Thin Film Electrocatalysts: Trade-Off between Specific Activity and Microscopic Porosity. *ACS Applied Materials & Interfaces* **2024**, *16* (26), 33620-33632. DOI: 10.1021/acsami.4c06308.
